# Mystery Solved: The Identification of the Two Missing Romanov Children Using DNA Analysis

**DOI:** 10.1371/journal.pone.0004838

**Published:** 2009-03-11

**Authors:** Michael D. Coble, Odile M. Loreille, Mark J. Wadhams, Suni M. Edson, Kerry Maynard, Carna E. Meyer, Harald Niederstätter, Cordula Berger, Burkhard Berger, Anthony B. Falsetti, Peter Gill, Walther Parson, Louis N. Finelli

**Affiliations:** 1 Armed Forces DNA Identification Laboratory, Armed Forces Institute of Pathology, Rockville, Maryland, United States of America; 2 Institute of Legal Medicine, Innsbruck Medical University, Innsbruck, Austria; 3 University of Florida, Gainesville, Florida, United States of America; 4 Department of Pure and Applied Chemistry, University of Strathclyde, Glasgow, United Kingdom; 5 Institute of Forensic Medicine, University of Oslo, Oslo, Norway; Max Planck Institute for Evolutionary Anthropology, Germany

## Abstract

One of the greatest mysteries for most of the twentieth century was the fate of the Romanov family, the last Russian monarchy. Following the abdication of Tsar Nicholas II, he and his wife, Alexandra, and their five children were eventually exiled to the city of Yekaterinburg. The family, along with four loyal members of their staff, was held captive by members of the Ural Soviet. According to historical reports, in the early morning hours of July 17, 1918 the entire family along with four loyal members of their staff was executed by a firing squad. After a failed attempt to dispose of the remains in an abandoned mine shaft, the bodies were transported to an open field only a few kilometers from the mine shaft. Nine members of the group were buried in one mass grave while two of the children were buried in a separate grave. With the official discovery of the larger mass grave in 1991, and subsequent DNA testing to confirm the identities of the Tsar, the Tsarina, and three of their daughters – doubt persisted that these remains were in fact those of the Romanov family. In the summer of 2007, a group of amateur archeologists discovered a collection of remains from the second grave approximately 70 meters from the larger grave. We report forensic DNA testing on the remains discovered in 2007 using mitochondrial DNA (mtDNA), autosomal STR, and Y- STR testing. Combined with additional DNA testing of material from the 1991 grave, we have virtually irrefutable evidence that the two individuals recovered from the 2007 grave are the two missing children of the Romanov family: the Tsarevich Alexei and one of his sisters.

## Introduction

For over 300 years, the Romanovs ruled the country of Russia. In 1917 following the Bolshevik revolution, the last ruling Russian Tsar, Nicholas II, abdicated his crown in favor of his brother Grand Duke Michael, who declined to accept the throne. Nicholas and his family - his wife, the Tsarina Alexandra, and their five children: Olga, Tatiana, Maria, Anastasia, and the Tsarevich (Crown Prince) Alexei were held in exile in Yekaterinburg, Russia. Also present with the royal family were four loyal members of their staff: Dr. Eugene Botkin, the family physician; Alexei Trupp, valet to the Tsar; Anna Demidova, maid to the Tsarina; and Ivan Kharitonov, the family cook.

In July of 1918, the Ural Soviets feared an attempt to rescue the Tsar and his family by the White Russian Army [1]. A decision was made by the Ural Soviets to execute the entire family, with the idea that upon hearing of the Tsar's death the will of the people loyal to the Tsar would be broken. In the early morning hours of July 17, 1918 the royal family and their staff were led to the cellar of the Ipatiev House where they were being held and executed.

In the late 1970s, a local geologist, Dr. Alexander Avdonin was able to locate the mass grave containing the remains of five of the seven members of the royal family and their four servants. Avdonin and a handful of close friends kept the location of the grave a secret until the fall of the Soviet Union in 1991 [2].

An official recovery and forensic anthropological investigation was conducted on the nine skeletons disinterred from the mass grave. DNA testing of the remains recovered in 1991 was conducted by Dr. Peter Gill, formerly of the Forensic Science Service (FSS) and Dr. Pavel Ivanov, a Russian geneticist [3]. Nuclear DNA testing of five STR markers confirmed the sex of the skeletons and established a familial relationship among the remains of the Tsar, the Tsarina and three of their daughters recovered from the grave. Previous mtDNA testing (outlined in Figure S1) confirmed a maternal relationship between HRH Prince Philip, the Duke of Edinburgh, the Tsarina, and her three daughters. The Duke of Fife and Princess Xenia Cheremeteff Sfiri, maternal relatives of Nicholas were used to reassociate the putative remains of the Tsar. A single point heteroplasmy at position 16169 (C/T = “Y”) was observed in the mtDNA sequence of the Tsar, whereas his maternal relatives were fixed for 16169 T. To confirm the authenticity of the heteroplasmy, DNA testing conducted at the Armed Forces DNA Identification Laboratory (AFDIL) compared the mtDNA haplotype from the remains of Grand Duke Georgij Romanov (d. 1899), brother of Tsar Nicholas II [4]. Both Tsar Nicholas II and Grand Duke Georgij Romanov shared the same point heteroplasmy at 16169 – but in differing ratios. The Tsar was mostly C/t while his brother was mostly T/c.

Despite the overwhelming forensic evidence, doubts pertaining to the authenticity of the remains persisted [5]. Skeptics pointed to the two children missing from the mass grave - Alexei and one of his sisters - as evidence that the bodies found in the mass grave were not the Romanov family. The identity of the missing princess was the source of a high profile disagreement between Russian and US forensic anthropologists: the Russians were convinced that Maria was missing from the mass grave, while the American experts believed that Anastasia was missing [2]. Rather than bring closure to the nearly 70 year mystery of the fate of the Romanovs, identification of only five of the seven family members continued to fuel speculation that somehow these two miraculously escaped the bullets of the executioners and made their way out of Russia.

After the discovery of the “first” mass grave, several attempts were made in the ensuing years to find the “second” grave, which was believed to be relatively nearby (P. Sarandinaki, personal communication). In the summer of 2007, a group of amateur archeologists discovered a few bone fragments approximately 70 meters from the first grave. Following an official archeological excavation conducted by Dr. Sergei Pogorelov, Deputy Director of the Sverdlovsk Region's Archaeological Institute, a set of 44 bone fragments and teeth were carefully recovered from the site.

After a thorough analysis of the remains by both Russian and US anthropologists, the scientific conclusions were the following:

- Based on duplicative anatomical units such as the midline portion of the occipital, no less than, or a minimum of two people were present among the recovered remains.
- One person present among the remains was of female sex, based on clearly visible sciatic notch dimensions, with a biological or developmental age of approximately 15–19 years.
- The sex of the other person was probably male, again based on the incipient breadth of the sciatic notch, and the biological age ranged from 12–15 years.
- Given the limited fragmented material coupled with the lack of representative diagnostic anatomy, it was not possible to determine the racial or ancestral type or estimate living stature from the remains.
- Three silver amalgam fillings discovered on the crowns of two molars recovered from the grave suggest that at least one person was of an aristocratic status.
- The overall age of the burial site was most likely greater than 60 years old based on culturally diagnostic material found contextually with the bones.

In late 2007, the Russian government invited a team of scientists to conduct independent DNA testing of the remains from the second grave. We present the results from mtDNA, autosomal STR and Y-STR testing of these remains at two independent laboratories highly specialized in ancient DNA (aDNA) studies: the Armed Forces DNA Identification Laboratory (AFDIL, Rockville, Maryland, USA) and the Institute for Legal Medicine (GMI, Innsbruck, Austria). We also present the results of a new analysis of the remains from the first mass grave attributed to Tsar Nicholas II, his wife Alexandra, Olga, Tatiana and a third daughter who could be either Anastasia or Maria. The DNA analysis of all three genetic systems confirms that the samples tested from the second grave are one female and one male child of Tsar Nicholas II and Tsarina Alexandra, solving the mystery of the missing Romanov children.

## Results

### Quantification

DNA quantification gave values greater than 4000 mitochondrial genome equivalent per microliter (mtGEs/µl) for the 143 bp mtDNA target except for bone sample 4.44 which contained less than 100 mtGEs/µl and bone sample 5.21 which contained 2923 mtGEs/µl (details in Table S1). No indication of the presence of PCR inhibitors was observed. Quantification of nuclear DNA produced concentrations between 11 and 615 pg/µl for all samples. No detectable quantification results were observed in any reagent blank control for either the mtDNA or the nuclear DNA targets.

### Mitochondrial DNA Testing

We first analyzed the remains discovered in 2007 (Table 1). We obtained full control region profiles [16024-576] for three samples (144.1, 146.1 and 147). Sequences of all three samples between 16024 and 576 were confirmed by three independent teams to be: 16111T, 16357C, 16519C, 263G, 315.1C, 524.1A and 524.2C. The common 16519 C variant and an AC doublet insertion in the HVIII AC repeat region are newly characterized variants outside of HVI/HVII for these samples compared to the original mtDNA testing [3] where these regions were not sequenced. For samples 140, 141, 143 and 145, we analyzed HVI [16024-16391] and HVII [35-369]. Samples sequenced for HVI and HVII were confirmed for 16111T, 16357C, 263G, and 315.1C. Two samples (139 and 142) failed to yield reproducible data.

**Table 1:** Sequences of the samples recovered from “Grave #2” in August 2007 and tested in this study.

Bone	Russian #	Region Sequenced	Sequence
Right humerus	141	16024-16391 and 35-369	16111T, 16357C, 263G, 315.1C
Occipital fragment	139	no results	–
Occipital fragment	144.1	16024-576	16111T, 16357C, 16519C, 263G, 315.1C, 524.1A; 524.2C
Right os coxae-♀	145	16024-16391 and 35-369	16111T, 16357C, 263G, 315.1C
Left femur	146.1*	16024-576	16111T, 16357C, 16519C, 263G, 315.1C, 524.1A; 524.2C
Right femur - ♀	147*	16024-576	16111T, 16357C, 16519C, 263G, 315.1C, 524.1A; 524.2C
Right scapula	140	16024-16391 and 35-369	16111T, 16357C, 263G, 315.1C
Cranial fragment	143	16024-16391 and 35-369	16111T, 16357C, 263G, 315.1C
Left ilium	142	no results	–

Samples marked with an asterisk (*) were tested by AFDIL and GMI.

Second, full control region [16024-576] profiles were generated from the remains of the Tsarina and three of her daughters recovered originally from the first grave (Table 2). Successful amplifications were obtained for all skeletal elements, with amplicons as large as 444 bp (GMI) or 440 bp (AFDIL). Sequences from all individuals confirmed previously published results in HVI and HVII and matched the sequence haplotype obtained with the recently discovered skeletal remains [3].

**Table 2:** Samples recovered from “Grave #1” in the early 1990s and tested in this study.

Skeleton	Attribution	Samples	Bone/Teeth
# 3	Olga	3.46*	Fragment of a left femur
		3.4	Partial tooth
#4	Nicholas	4.29	Fragment of a rib
		4.51*	Fragment of a calcaneus
		4a*	Partial tooth
		*4.44*	*Fragment of a pelvis*
#5	Tatiana	5.21*	Fragment of a left femur
		5.29	Fragment calcaneus
#6	Anastasia	6.14*	Fragment of the diaphyse of a left femur
		6.16*	Fragment of the diaphyse of a left tibia
#7	Alexandra	7.48	Fragment of a pelvis
		7.49*	Fragment of the diaphyse of a left tibia
		7a	Partial tooth
		*7.40*	*Fragment of the diaphyse of a left femur*

Samples marked with an asterisk (*) were sent to both AFDIL and GMI for testing. Samples in italics were sent only to GMI. All other samples were tested by AFDIL.

To assess the frequency of this sequence, we first focused on the German database (n = 513 samples) within the EDNAP mtDNA Population Database (EMPOP; [6]) since Tsarina Alexandra was a German princess. We found no exact matches either in the German database or within the 3,340 West Eurasia sequences in EMPOP. Finally, we searched the Tsarina's haplotype using a global mtDNA database of 23,627 individuals (4,839 individuals in the US SWGDAM mtDNA database and 18,788 individuals from an internal AFDIL Research Section database as of 01/16/09) and found no match making this haplotype a rare sequence.

**Figure 1:**
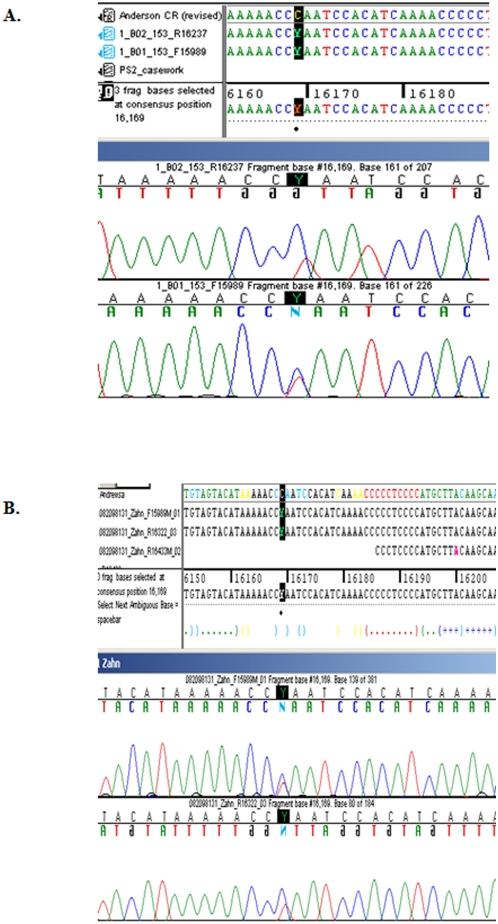
Screenshot of the 16169 C/T heteroplasmy present in Tsar Nicholas II using both forward and reverse sequencing primers. (A) Results from AFDIL's casework section. (B) Results from GMI.

The sequence of the full control region [16024-576] was also determined from the remains of Tsar Nicholas II (a tooth from skeleton #4) and matched the published data of HVI and HVII from Gill *et al.* [3] and Ivanov *et al.* [4]: 16126C, 16169Y, 16294T, 16296T, 16519C, 73G, 263G, 315.1C with the transition at position 16519 newly characterized in the control region. The point heteroplasmy at position 16169 was present with C being the major component over T (Figure 1).

To assess the frequency of the haplotype from the Tsar, we first focused on the Denmark database (n = 209 samples) within the EMPOP database since the Tsar's mother was a Danish princess. We found no exact matches in the Danish database. Among the 3,340 West Eurasian sequences in EMPOP, 3 sequences matched (0.09%) when 16169C (matching the rCRS) was considered. A search of the AFDIL+SWGDAM mtDNA database revealed 19 matches (19/23,627 = 0.08%) to the Tsar's haplotype when 16169C was considered. No matches to the Tsar's haplotype+16169T were observed. The relative frequency of the Tsar's mtDNA haplotype was considered to be rare.

### Autosomal STR Testing

The best preserved fragments were identified by an anthropological inspection and two compact bone fragments, each coming from a femoral bone (146.1 and 147), were selected for nuclear STR testing. An anthropologist (ABF) determined that fragment 147 likely belonged to a female based on the general size and shape of the femoral head and angulations of the femoral neck.

The STR results for samples 146.1 and 147 are shown in Table 3. Each allele was replicated at least seven times by both laboratories. The results of the sex-typing marker amelogenin revealed that sample 146.1 was from a male and confirmed that sample 147 was from a female (Figures S2 and S3). We found no evidence of contamination among our STR profiles. In fact, of the 1458 alleles amplified above our reporting threshold at AFDIL (100 RFUs for heterozygote alleles and 200 RFUs for homozygous alleles) among all autosomal and Y-STRs, only 6 alleles (0.4%) would be considered as spurious or drop-in artifacts. Half of these spurious alleles occurred at stutter positions of the authentic allele – indicating that they were most likely generated by preferential amplification during the early rounds of PCR.

**Table 3:** Autosomal STR Genotypes for the Romanov Family.

Marker	Sample 4.3	Sample 7.4	Sample 3.46	Sample 5.21	Sample 6.14	Sample 147	Sample 146.1
	Tsar Nicholas II	Tsarina Alexandra	Olga	Tatiana	Maria or Anastasia	Anastasia or Maria	Alexei
Amelog	X, Y	X, X	X, X	X, X	X, X	X, X	X, Y
D3S1358	14, 17	16, 18	17, 18	17, 18	16, 17	17, 18	14, 18
TH01	7, 9.3	8, 8	8, 9.3	7, 8	8, 9.3	7, 8	8, 9.3
D21S11	32.2, 33.2	30, 32.2	30, 33.2	32.2, 33.2	30, 33.2	30, 33.2	32.2, 33.2
D18S51	12, 17	12, 13	12, 12	12, 12	13, 17	12, 17	12, 17
D5S818	12, 12	12, 12	12, 12	12, 12	12, 12	12, 12	12, 12
D13S317	11, 12	11, 11	11, 11	11, 11	11, 11	11, 11	11, 12
D7S820	12, 12	10, 12	12, 12	10, 12	12, 12	10, 12	12, 12
D16S539	11, 14	9, 11	11, 11	11, 11	11, 14	9, 11	11, 14
CSF1PO	10, 12	11, 12	11, 12	11, 12	10, 11	10, 12	10, 12
D2S1338	17, 25	19, 23	17, 19	23, 25	17, 19	17, 23	23, 25
vWA	15, 16	15, 16	15, 16	15, 16	15, 16	15, 16	15, 16
D8S1179	13, 15	16, 16	13, 16	15, 16	13, 16	15, 16	15, 16
TPOX	8, 8	8, 8	8, 8	8, 8	8, 8	8, 8	8, 8
FGA	20, 22	20, 20	20, 22	20, 20	20, 22	20, 22	20, 22
D19S433	13, 13.2	13, 16.2	13.2, 16.2	13.2, 16.2	13, 16.2	13, 13	13, 13.2

The STR analysis revealed the presence of two separate individuals and a very high degree of allele sharing was noted among the two profiles, suggesting that the individuals were closely related. The Sibship Index (SI) was calculated by determining the likelihood ratio (LR) of the hypothesis (H_1_) that samples 146.1 and 147 are siblings compared to the alternative hypothesis that these samples belong to two unrelated individuals (H_2_). The SI was determined to be 5.6 million in favor of H_1_. In other words, the DNA evidence is 5.6 million times more likely if samples 146.1 and 147 were siblings rather than if these samples were from two unrelated individuals.

To confirm that these two siblings were also related to the Romanov remains recovered from the first mass grave, we conducted STR testing on skeletal elements representing the other five members of the royal family (Table 2). The results are shown in Table 3. The profiles for vWA matched the data published by Gill *et al.* [3] from the quadruplex markers tested [7]. For the TH01 locus, we obtained alleles 7 and 9.3 for the Tsar, identified as a 7/10 genotype from Gill *et al.* [3]. Similarly, for one of the daughters, the TH01 genotype was previously 8/10 and is now 8/9.3. In the Gill *et al.* publication [3], it was the practice at the time to combine the 9.3 and 10 alleles for TH01 and to use the ‘10’ designation as the standard nomenclature for both. The DNA Commission of the International Society for Forensic Haemogenetics (ISFH) later recommended the use of “9.3” to describe the microvariant allele in TH01 [8]. After the year 2000, the 9.3 allele designation at TH01 was utilized for all profiles sent to the UK national DNA database. This minor (historical) difference in the nomenclature has no effect on our comparisons. All of the genotypes at vWA and TH01 were fully concordant among the skeletons from the first grave.

All of the additional microsatellites tested in this study confirmed the parental relationship between the skeletal remains of Tsar Nicholas II and Alexandra and the other remains tested in this study. All of the alleles from three daughters from the first grave can be explained by half-allele sharing with the profiles from Nicholas and Alexandra. Importantly, the two skeletal remains from the newly discovered grave show the same half allele sharing genotypes with both Nicholas and Alexandra as putative parents. When we calculated the LR of the hypothesis (H_1_) that samples 146.1 and 147 are the children of Tsar Nicholas II and Tsarina Alexandra (and siblings of the three princesses from grave one) compared to the alternative hypothesis that these samples are individuals completely unrelated to the Romanov family (H_2_), we found that the DNA evidence is 4.36 trillion times more likely if sample 147 is a daughter of Tsar Nicholas II and Tsarina Alexandra, and over 80 trillion times more likely if sample 146.1 is a son of Tsar Nicholas II and Tsarina Alexandra than if these samples were from two unrelated individuals.

### Y-STR Testing

**Figure 2:**
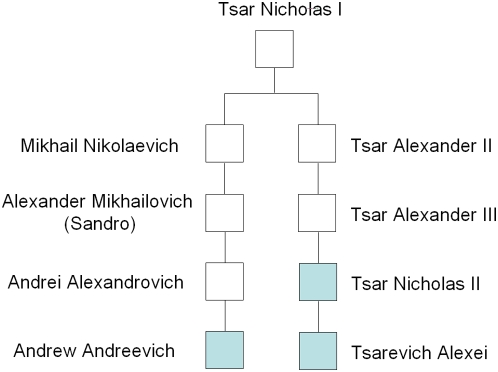
Romanov paternal lineage used for Y-STR testing. DNA testing for 17 Y-STR markers was conducted on the remains from Tsar Nicholas II and his son, the Tsarevich Alexei (sample 146.1 in the second grave). A distantly related cousin, Prince Andrew Andreevich Romanov of San Francisco, California, was used as a living relative to compare to the skeletal material.

**Figure 3:**
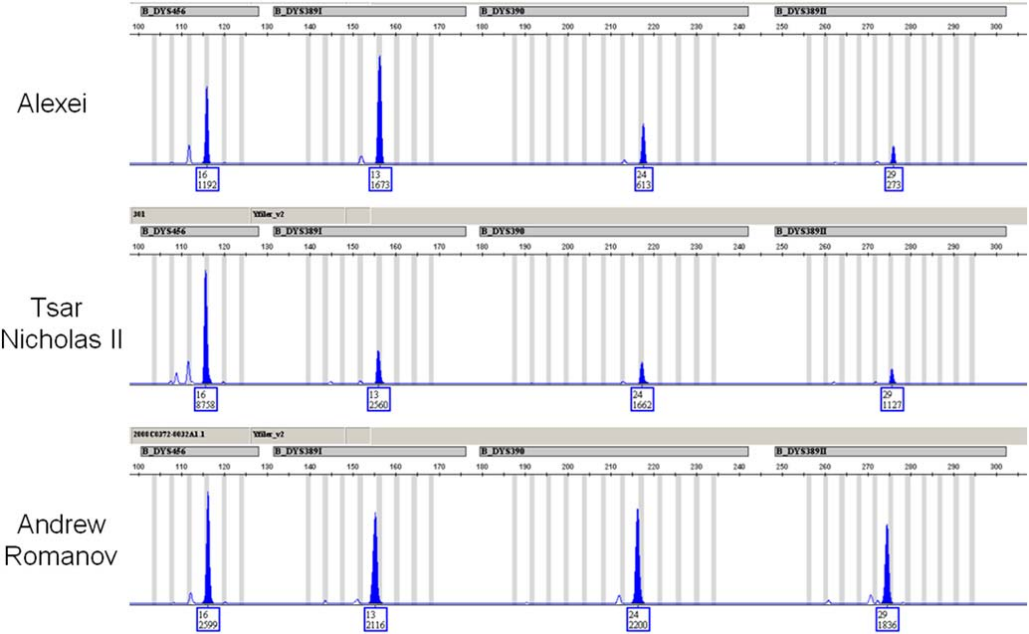
An example of four Y-STR markers from the three Romanov relatives. Each panel is a screenshot from the blue dye channel of Y-Filer (Applied Biosystems, Foster City, CA). The top panel was developed from the skeletal remains of Alexei, the middle panel was developed from a tooth sample from Tsar Nicholas II, and the bottom panel was developed from Prince Andrew Andreevich Romanov. The loci are (from L to R): DYS456 (16 repeats), DYS389I (13 repeats), DYS390 (24 repeats), and DYS389II (29 repeats).

Finally, to compare the profile of the Tsar and his son to a paternally living descendant of the Romanov family, we conducted Y-STR testing on the skeletal material. We first generated a 17 Y-STR loci profile from sample 146.1 and then from a tooth of the Tsar. Finally, in a separate laboratory, we generated the profile of Prince Andrew Andreevich Romanov, a distantly related cousin of Tsar Nicholas II (Figure 2). An example of four of the markers is shown in Figure 3. We observed an exact match between all three men over all 17 markers (Table 4). To determine the significance of this match, we searched the Y-STR haplotype against a database of 4,163 individuals (http://usystrdatabase.org/) and found no match. A search of the YHRD database (http://www.yhrd.org) was conducted and no match was observed between the 17 locus profile and the 10,243 haplotypes including at least 2,068 individuals from the Eurasian Metapopulation.

**Table 4:** Y-STR haplotype for Nicholas, Alexei and Andrew Romanov.

DYS19	DYS389I	DYS389II	DYS390	DYS391	DYS392	DYS393	DYS385a/b
14	13	29	24	10	13	13	11, 14
DYS438	DYS439	DYS437	DYS448	DYS456	DYS458	DYS635	YGATAH4
12	11	15	19	16	17	24	12

## Discussion

The true fate of the Romanov family was unknown to all except for a handful of people for nearly 70 years. Gill *et al.* [3] conducted the original DNA testing after the preliminary anthropological investigations from the first grave.

**Figure 4:**
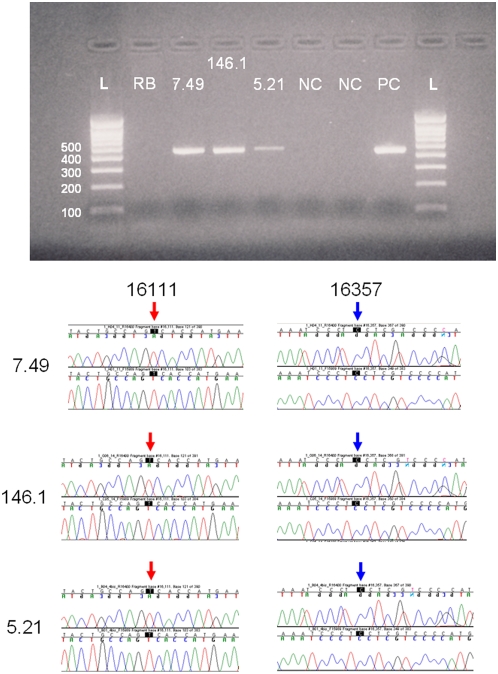
Screenshots of the 16111 C-T variant and 16357 T-C variants from three samples sequenced from fragments amplified for 440 bp. Legend for the gel: L = Ladder, RB = Reagent Blank, 7.49 = Sample from Tsarina Alexandra, 146.1 = Sample from Alexei, 5.21 = Sample from Tatiana, NC = Negative Control, PC = Positive Control, L = Ladder.

The veracity of the results were later challenged by Knight *et al.* [9] who doubted the authenticity of sequences generated from the nested PCR strategy. Knight argued that the amplicon sizes were unusually long, and therefore the results were unreliable. Hofreiter *et al.* [10] and Gill and Hagelberg [11] have offered a rebuttal to the opinions made by Knight *et al.* [9]. However, Knight *et al.* [12] insisted: “To the contrary, only a 221-bp amplicon could be produced (possibly from endogenous degraded DNA template), but not a 400-bp nested product…. [the] results in (Gill *et al.*) are not plausible”. It is generally our experience that highly degraded mtDNA templates are often only amplified with 270 bp amplicons or less. However, given this unique opportunity to re-test the exact material originally evaluated by Gill *et al.* [3], we were successfully able to amplify 444 bp and 440 bp fragments using a classic aDNA amplification strategy (increased cycle number, additional BSA, and additional polymerase), see Figure 4. Not only was it possible to amplify up to 444 bp of mtDNA, we also successfully amplified a number of high molecular weight alleles from the nuclear STRs tested (under ∼375 bp for autosomal markers and under ∼335 bp for the Y-chromosomal markers). It is very likely that the extremely cold climate in Yekaterinburg, where the ground is typically frozen from September until April, provided an ideal environment to preserve the remains.

Another issue which generated doubt about the early DNA testing was the point heteroplasmy at np 16169 in the mtDNA sequence of the Tsar. At the time this was a contentious finding. In the early to mid-1990s, point heteroplasmy was believed to be an extremely rare phenomenon and was not easily explained as the presence of two different mtDNA haplotypes within an individual. Independent testing of samples was extremely important to provide the necessary confidence that the results were valid. The mtDNA results from the alleged Tsar were thus independently confirmed in the laboratory of Erika Hagelberg at Cambridge University, a co-author on the 1994 publication [3]. In addition, the 16169 T/C point heteroplasmy was confirmed by AFDIL in bone samples from Tsar Nicholas II and his brother, Grand Duke Georgij [4]. Finally the heteroplasmy was detected and confirmed once again in this study by both AFDIL and GMI (Figure 1).

Today, the existence of heteroplasmy is understood to be relatively common, although occurrence at the specific 16169 position is itself rare [13]. Among our internal AFDIL mtDNA database of 18,788 haplotypes, we have observed three instances (0.016%) of point heteroplasmy at np 16169. Only one sample of Western European ancestry shares the same haplogroup T* as the Tsar, but differs from Nicholas at seven nucleotides within the control region. Consequently, multiple observations of this rare heteroplasmic event can be considered to be a very powerful indicator of relatedness.

Two questions posed by Gilbert *et al.* [14] to assess the results of an aDNA study is for the researcher, reader, and reviewer to ask the questions, “What information is presented here that makes the results and/or conclusions believable?” and “Is there any reason to not believe this?” We used a three-pronged DNA marker system to develop our results.

The ***mtDNA*** results alone can be considered conclusive. The new samples matched exactly the mtDNA data of Tsarina Alexandra (and the HVI and HVII data of a living relative, HRH Prince Philip), indicating that these samples were maternally related to her. If one includes the anthropological information about these samples: specifically that one of the samples recovered from the second grave was most likely the femur of a young woman (sample 147), we can conclude that these samples were from the missing children of the Tsarina since the femora from the Tsarina and her three other children were recovered and accounted for in the first grave.

***Autosomal STR*** genotypes were developed to form a family pedigree of the Romanov family. The DNA profiles of the two samples from the second grave fit perfectly into the family tree of the Tsar and Tsarina with all of the alleles of the two samples explained by Mendelian inheritance.

A 17-marker ***Y-STR*** haplotype from the remains of Tsar Nicholas II matched exactly to the Y-STR haplotype from femur of the male sample (sample 146.1) found in the second grave. The same 17-marker haplotype was also observed to match a living Romanov relative.

After examining mitochondrial sequences, autosomal STR and Y-STR profiles all linking the remains to living relatives of the Romanov, we also compared our STR results from Tsar Nicholas II with a profile developed by the Sverdlovsk Regional Forensic Bureau (Yekaterinburg) from a blood stain on a shirt worn by Nicholas when he was a young man. On April 29, 1891 while touring the city of Otsu, Japan the Tsarevich Nicholas Romanov was attacked by a Japanese policeman during an attempted assassination [15]. Nicholas sustained two blows to the side of his head from a saber used by the attacker before the assailant was subdued. Fortunately, Nicholas survived the attack and the bloody shirt he wore that day was returned to Russia as a relic of the attack. Eventually, the shirt was placed in storage at the Hermitage Museum in St. Petersburg. In the summer of 2008, the Sverdlovsk Regional Forensic Bureau took three samples from blood stains on the shirt. One of the samples gave full autosomal and Y-STR profiles [16]. The other two samples gave partial DNA profiles for both autosomal and Y-STRs, with all of the alleles in the partial profile shared with the alleles from the full profile. We compared our DNA profiles from the tooth of Nicholas II to the blood stain profile and found complete concordance at all loci. For the first time, there is now a link between the ante-mortem evidence DNA profile from Nicholas II to the post-mortem skeletal remains from the first grave.

Taken together, all of the results and conclusions agree with the hypothesis that the samples recovered from grave two are the missing children of Tsar Nicholas II and Tsarina Alexandra. It should be mentioned that a well publicized debate [2] over which daughter, Maria (according to Russian experts) or Anastasia (according to US experts), has been recovered from the second grave cannot be settled based upon the DNA results reported here. In the absence of a DNA reference from each sister, we can only conclusively identify Alexei – the only son of Nicholas and Alexandra.

For nearly ninety years the fate of the Romanov family was shrouded in mystery. It wasn't until several years after the execution of the family that the Soviet government acknowledged the death of all of the Romanovs. Speculation grew that some of the family escaped the executioners and found their way out of Russia. The most famous claimant was Anna Anderson, a Polish peasant who convinced many that she was Anastasia [2]. With the discovery of the first grave, and subsequent DNA testing, Anna Anderson was exposed as an imposter [17]. In fact, since 1918 over 200 people have claimed to be one of the five Romanov children (http://www.romanov-memorial.com/pretenders.htm). Here we are able to give a full account of all of the Romanov family and can conclude that none of the family survived the execution in the early morning hours of July 17, 1918.

### Materials Tested

#### Material from the grave discovered in 2007

Fragments from ten samples out of 44 were selected for DNA analysis: nine bone fragments (cranial, pelvic, scapular, or femoral) and one-half of the crown portion of a molar. It was determined that the tooth fragment would likely produce a DNA profile; however, we decided to preserve the material rather than destroy it during testing. Two of the nine samples (146.1 and 147) were divided and analyzed by 3 independent teams: the AFDIL (research section), the AFDIL (mitochondrial casework section) and the GMI laboratory. The mitochondrial casework section also analyzed the remaining seven samples and focused only on mtDNA testing for these samples following their standard operational protocols.

#### Material from the grave excavated in 1991

The remains of the Tsar and his family were laid to rest at the Cathedral of Saint Peter and Saint Paul in St. Petersburg, Russia in 2001. Fortunately, the Sverdlovsk Regional Forensic Bureau Laboratory (Yekaterinburg) anticipated the possibility of future DNA testing, and preserved a limited number of fragments from each skeleton. At least two to three samples per individual (bone and/or teeth) were brought to the AFDIL and to the GMI laboratories for DNA analysis. For convention, we followed the naming of the skeletons of the royal family according to the Russian anthropological/facial reconstruction studies from the mid-1990s: Skeleton #3 = Olga; Skeleton #4 = Tsar Nicholas II; Skeleton #5 = Tatiana; Skeleton #6 = Anastasia (or Maria); and Skelton #7 = Tsarina Alexandra (Table 2).

## Methods

DNA extractions and analysis were performed by three independent teams, all specialized in aDNA studies and working in adequate facilities. Specialists from the mitochondrial casework section of the AFDIL (MJW, SME, KM) worked in an American Society of Crime Lab Directors (ASCLD) accredited laboratory and focused on mitochondrial DNA analysis, following their standard operating protocols. One specialist from the research section (OML) used a separate laboratory devoted to aDNA studies and focused on STR analysis. Finally, the GMI team (HN, CB, BB) used an ISO 17025 accredited laboratory to replicate mtDNA and STR analysis. In all laboratories, precautions to monitor contamination by using controls throughout the process and isolation of pre-and post PCR areas were observed at all times. Material and equipment were cleaned using a 10% bleach solution and UV irradiated at 254 nm in a cross-linker for 10 to 45 minutes.

Experiments performed in all three laboratories were witnessed by two Russian scientists from the Sverdlovsk Regional Forensic Bureau Laboratory (Yekaterinburg): Tamara Tsitovich and Natalia Bandurenko at AFDIL; and Elena Trynova and Elena Vylegzhanina at GMI.

### Preparation of the samples

At the AFDIL, all the samples were first extensively sanded with an aluminum oxide sanding stone attached to a dremel tool (Dremel, Racine, WI), sonicated in DNA free water and absolute ethanol then placed in a sterilized fume hood to air-dry overnight. The next day, the samples were powdered with a cleaned, DNA-free stainless steel Waring MC2 blender cup (Waring, Torrington, CT).

At the GMI, sample pre-treatment comprised extensive mechanical cleaning of the surface of the bones and teeth with UV-irradiated sandpaper and/or sterile scalpel blades, followed by a 20 min soak in sodium hypochlorite solution (≥4% active chlorine), and one washing step each in sterile water and absolute ethanol. The cleaned samples were dried in a laminar flow hood under a constant air-stream over night and a final 15 min UV-irradiation step was applied. The dried samples were powdered by means of a sterile dental drill.

### DNA extractions

The GMI group and the mtDNA casework section extracted the DNA according to Loreille *et al.* [18] but the GMI team added a final purification step using the QIAquick PCR purification kit (Qiagen, Hilden, Germany) according to the manufacturer's instructions. The AFDIL research scientist used a slightly modified protocol that avoids organic extraction [19]. Between 100 and 400 mg of fine bone powder as well as a “reagent blank” were incubated and gently shaken in 3 ml of extraction buffer (EDTA 0.5 M, 0.5% lauryl-sarcosinate) and 100 µl of proteinase K (20 mg/ml) overnight at 56°C. The tubes were centrifuged for 3 minutes at 4000 g, the extraction buffer transferred into a Centricon 30 (Millipore Corp., Bedford, MA) and then concentrated until the volume had decreased to 100 ul. The solution was transferred into a clean tube and purified using the MinElute PCR Purification Kit (Qiagen, Valencia, CA). The final volume varied between 25 µl and 100 µl.

### DNA quantification

The mitochondrial DNA content of each sample tested at the GMI was determined by quantitative real-time PCR following the protocol detailed in Niederstätter *et al.* [20]. Nuclear DNA was quantified using the human-specific AluYb8 assay described in Walker *et al.* [21], including an internal amplification positive control to test for the presence of PCR inhibitors in the DNA extracts.

### Mitochondrial DNA analysis

At the GMI, five overlapping “midi”-amplicons ranging from 282 to 444 bp [22] in two multiplex PCR assays and carefully selected “mini”-amplicons [23] were amplified and sequenced with the PCR primers to generate a consensus sequence displaying full double-strand sequence coverage of the mtDNA control region. Sequencing was performed according to Berger and Parson [22] and Eichmann and Parson [23].

The AFDIL casework group amplified mtDNA using a redundant amplification strategy described in Edson *et al.* [24]. Hypervariable Regions one (HVI), two (HVII), and three (HVIII) as well as mini-variable region one (MVR1) were amplified for each sample as template availability and quality allowed. The size of the amplicons varied from 126 to 440 bp. All post-PCR products, including controls, were purified using ExoSAP-IT™ (USB Co., Cleveland, OH) and the purified templates were sequenced with the Big Dye Terminator Cycle sequencing kit (Applied Biosystems, Foster City, CA). Sequencing products were purified using Performa® DTR Ultra 96-well plates (Edge BioSystems, Gaithersburg, MD) and dried down in an evaporator/concentrator centrifuge. Formamide/EDTA (3∶1) was used to rehydrate the product prior to loading on an Applied Biosystems 3130*xl* Genetic Analyzer. The multiple sequences were aligned and compiled using the Sequencher software v4.7 (GeneCodes, Ann Arbor, Michigan). Differences from the revised Cambridge Reference Sequence [25], [26] were determined and used for mtDNA database searches.

### Autosomal and Y-STR analysis

STR testing was conducted with several standard commercially available kits. The GMI and the AFDIL both used the AmpFlSTR Identifiler, AmpFlSTR MiniFiler, and AmpFlSTR Yfiler PCR amplification kits (Applied Biosystems). For the analysis of bone sample 147, GMI also used the AmpFlSTR SEFiler amplification kit (Applied Biosystems). The GMI followed the protocols recommended by the manufacturer and also tested AmpFlSTR Identifiler and AmpFlSTR Yfiler using 34 cycles. The AFDIL used a low copy number approach [27] with the AmpFlSTR Identifiler and AmpFlSTR Yfiler PCR amplification kits and used twice the recommended Ampli*Taq* concentration and six additional PCR cycles [18], [28]. PCR amplification with the AmpFlSTR MiniFiler kit at AFDIL was performed according to the manufacturer's protocol. When necessary, both AFDIL and GMI used markers from an in-house miniSTR assay [29], [30]. All STR and Y-STR amplification products were analyzed on either a 3100 or a 3130*xl* Genetic Analyzer (Applied Biosystems). Analysis of the data was performed using GeneScan software v3.7 and Genotyper v3.7NT or Genemapper® v3.2. Fragment sizing was performed by means of an internal size standard (GeneScan-500 LIZ) and the amplicons were compared with the provided allelic ladder (AmpFlSTR Identifiler, AmpFlSTR MiniFiler and AmpFlSTR Yfiler allelic ladders) for unambiguous allele designation.

### Data Analysis and Statistical Calculations

To preserve the independence of the testing performed at each laboratory; no data was transferred to either laboratory during the testing period. Once the testing was completed at AFDIL and the GMI, electropherograms of the data and tabulated results were sent to an independent scientist (PG) for confirmation and concordance of the data.

We evaluated the weight of the autosomal STR evidence using a likelihood ratio (LR) where two competing hypotheses are evaluated:
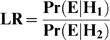



The likelihood approach above evaluates two competing scenarios. In the numerator, we evaluate the probability (Pr) of the DNA Evidence (E) given the hypothesis (H_1_) that the remains belong to the missing children of the Romanovs. In the denominator of the LR, we evaluate the probability of the DNA evidence given the alternative (null) hypothesis (H_2_) that these remains were not from the missing Romanov children, but were derived from two randomly sampled, unrelated individuals. The LR for autosomal STRs was calculated using the software program DNAView™ (Charles Brenner, Oakland, CA) through a customized interface developed for AFDIL called LISA (Laboratory Information Systems Application, FTI Inc., Fairfax, VA).

## Supporting Information

Figure S1 mtDNA lineage information of previous and present Romanov testing. *The identification of either Maria or Anastasia was not possible by DNA analysis alone. Either name could be interchangeable in this pedigree.
(2.03 MB TIF)

Figure S2 An example electropherogram using the MiniFiler STR kit for sample 146.1 (Alexei Romanov).
(2.35 MB TIF)

Figure S3 An example electropherogram using the MiniFiler STR kit for sample 147 (either Grand Duchess Maria or Anastasia Romanov).
(2.40 MB TIF)

Table S1 Quantification results of the samples tested at GMI. MtGE: mitochondrial genome equivalent. Samples 147 and 4.51 were both extracted twice independentely. The values shown in the table represent the DNA concentration of each extract.
(0.03 MB DOC)
