# Supplementary figures and images for: Mystery Solved: The Identification of the Two Missing Romanov Children Using DNA Analysis
Source: PLoS One. 2009 Mar 11;4(3):e4838. doi: 10.1371/journal.pone.0004838 (PMC2652717; doi:10.1371/journal.pone.0004838)

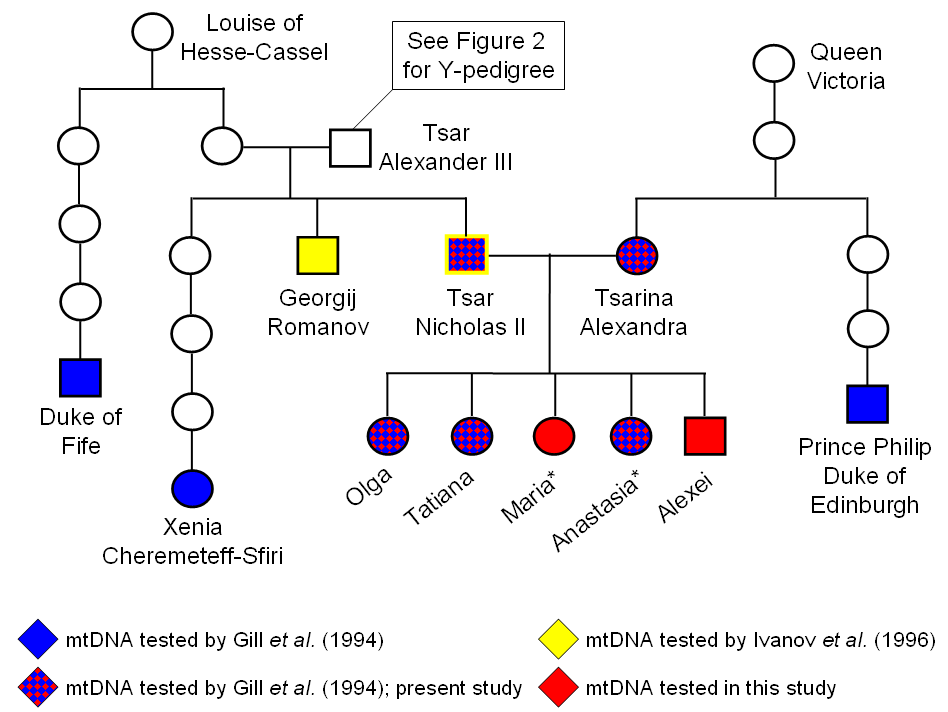

Supplement: Figure S1 — mtDNA lineage information of previous and present Romanov testing. *The identification of either Maria or Anastasia was not possible by DNA analysis alone. Either name could be interchangeable in this pedigree. (2.03 MB TIF) [file pone.0004838.s001.tif]

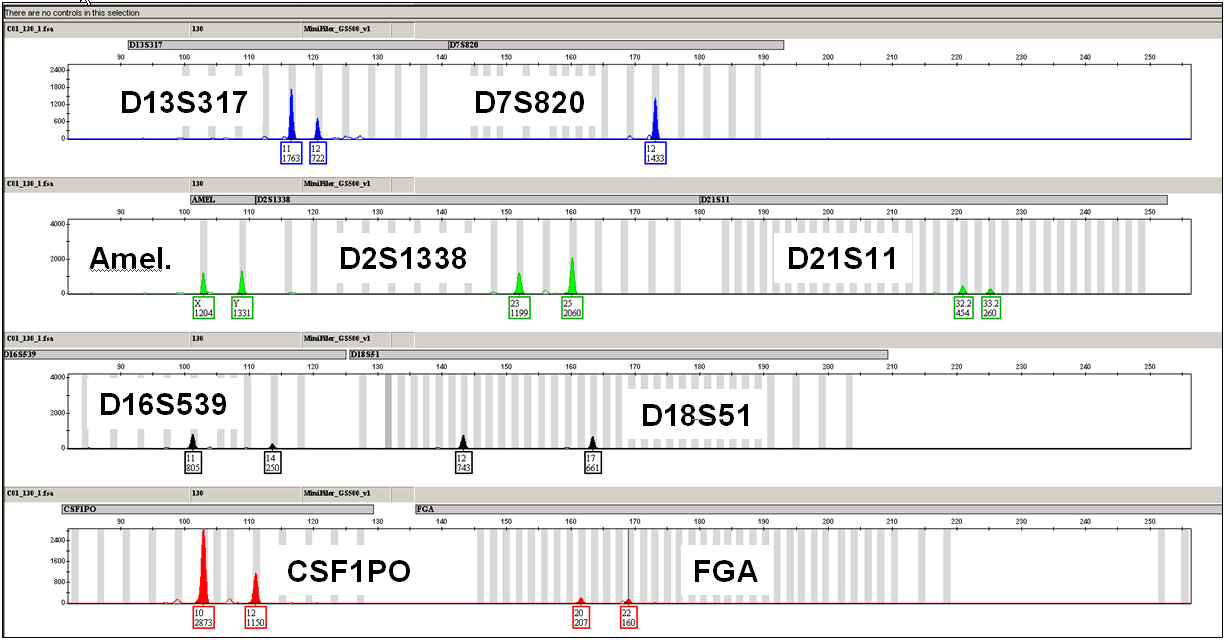

Supplement: Figure S2 — An example electropherogram using the MiniFiler STR kit for sample 146.1 (Alexei Romanov). (2.35 MB TIF) [file pone.0004838.s002.tif]

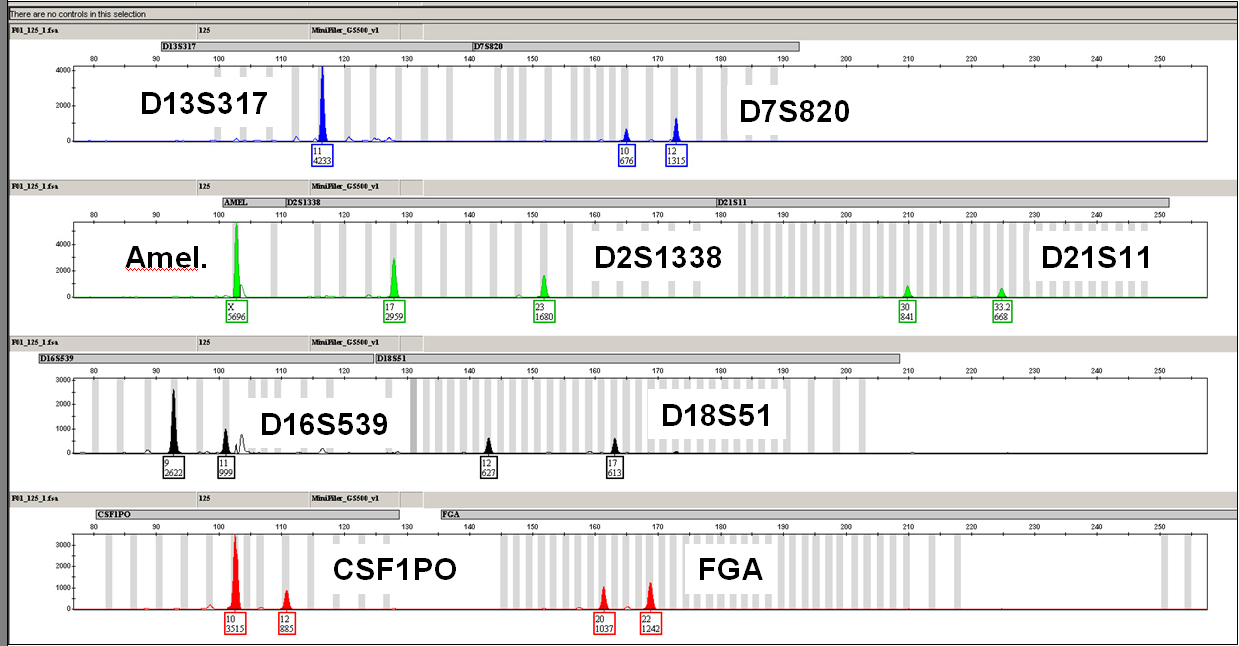

Supplement: Figure S3 — An example electropherogram using the MiniFiler STR kit for sample 147 (either Grand Duchess Maria or Anastasia Romanov). (2.40 MB TIF) [file pone.0004838.s003.tif]
