# Supplementary material for: Mystery Solved: The Identification of the Two Missing Romanov Children Using DNA Analysis
Source: PLoS One. 2009 Mar 11;4(3):e4838. doi: 10.1371/journal.pone.0004838 (PMC2652717; doi:10.1371/journal.pone.0004838)
Supplement: Table S1 — Quantification results of the samples tested at GMI. MtGE: mitochondrial genome equivalent. Samples 147 and 4.51 were both extracted twice independentely. The values shown in the table represent the DNA concentration of each extract. (0.03 MB DOC) [file pone.0004838.s004.doc]

**Table S1. Quantification results of the samples tested at GMI.**

| **Samples grave 2** | mtGE 143bp/μl | Qty AluYb8 (pg/μl) |
| --- | --- | --- |
| 146.1 (bone) | 9,707 | 16 |
| 147 (bone) | 4,934 / 4,239 | 37/ 29 |
| **Samples grave 1** |  |  |
| 3.46 (bone) | 60,291 | 186 |
| 4a (tooth) | 11,279 | 615 |
| 4.44 (bone) | <100 | 12 |
| 4.51 (bone) | 5,261/ 5,562 | 45/ 11 |
| 5.21 (bone) | 2,923 | 11 |
| 6.14 (bone) | 9,396 | 11 |
| 6.16 (bone) | 17,794 | 136 |
| 7.4 (bone) | 11,773 | 19 |
| 7.49 (bone) | 16,806 | 49 |

MtGE: mitochondrial genome equivalent. Samples 147 and 4.51 were both extracted twice independentely. The values shown in the table represent the DNA concentration of each extract.
